# Right Ventricular–Pulmonary Arterial Coupling and Outcome in Heart Failure With Preserved Ejection Fraction

**DOI:** 10.1002/clc.24308

**Published:** 2024-07-16

**Authors:** Jia Wang, Xiang Li, Jiahui Jiang, Ze Luo, Xilun Tan, Ruhua Ren, Tsutomu Fujita, Yoshifumi Kashima, Tetsuaki Li Tanimura, Ming Wang, Chenhao Zhang

**Affiliations:** ^1^ Emergency Department Wangjing Hospital of China Academy of Chinese Medical Sciences Beijing China; ^2^ Department of Cardiology Chongqing Hospital of Traditional Chinese Medicine Chongqing China; ^3^ College of Traditional Chinese Medicine Chongqing Medical University Chongqing China; ^4^ Department of Cardiology Sapporo Cardio Vascular Clinic Sapporo Japan

**Keywords:** echocardiography, heart failure, preserved ejection fraction, right ventricular–pulmonary arterial coupling

## Abstract

**Background:**

Right ventricular–pulmonary artery coupling (RVPAC) refers to the relationship between right ventricular systolic force and afterload. The ratio of echocardiograph‐derived tricuspid annular plane systolic excursion (TAPSE) to pulmonary artery systolic pressure (PASP) has been proposed as a noninvasive measurement of RVPAC and reported as an independent prognostic parameter of heart failure. However, it has not been adequately in detail evaluated in heart failure with preserved ejection fraction (HFpEF). We hypothesized that RVPAC may be used and proposed as an expression of key risk factors in patients with HFpEF.

**Methods:**

We retrospectively analyzed TAPSE/PASP of 648 HFpEF patients hospitalized in Chongqing Hospital of Traditional Chinese Medicine from January 1, 2016 to January 1, 2017. All eligible patients were followed up for 5 years. The correlation between TAPSE/SPAP index and clinical indicators and outcomes was evaluated.

**Results:**

The final analysis included 414 patients. Nonsurvivors had significantly lower TAPSE, TAPSE/PASP and higher PASP compared with survivors (*p* < 0.0001). ROC curve analysis showed that the optimal cutoff of TAPSE, PASP, and RVPAC to predict all‐cause death were 16.5 mm, 37.5 mmHg, and 0.45 mm/mmHg, respectively. In multivariate Cox regression analyses adjusted for gender showed a significant, independent association of the RVPAC with the composite endpoint of all‐cause death or HF‐related recurrent hospitalization (HR: 0.006; 95% CI 0.001–0.057, *p* < 0.001).

**Conclusions:**

RVPAC, defined by the ratio of TAPSE to PASP, is the expression of a key risk factor in HFpEF patients, which is independently associated with the composite endpoint of all‐cause death or HF‐related recurrent hospitalization.

## Graphical abstract

RVPAC, defined by the ratio of TAPSE to PASP, is the expression of a key risk factor in HFpEF, which is associated with poor outcomes. When the ratio < 0.45, the risk of all‐cause death or HF‐related readmission in HFpEF patients is increased.

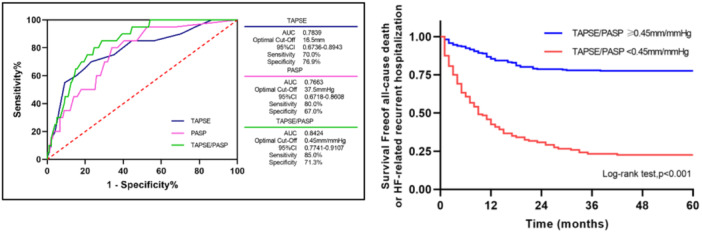

## 1 Introduction

Heart failure (HF) is a complex clinical syndrome with symptoms that result from any structural or functional impairment of ventricular filling or ejection of blood that can be divided into HF with a reduced ejection fraction (HFrEF, ≤ 40%) and HF with a preserved ejection fraction (HFpEF, ≥ 50%) according to the ejection fraction of left heart (LV) [1, 2, 3]. In the past, due to researchers' attention to HFrEF, the establishment of effective prevention and treatment strategies for HFrEF has reduced the incidence of HFrEF [4, 5]. In the aging population, HFpEF is becoming the main form of HF [6]. Patients with HFpEF have a devastating 5‐year mortality rate (approaching 60%), costly morbidity (6‐month hospitalization rate of 50%), and debilitating symptoms (reduced exercise capacity and maximum myocardial oxygen consumption [MVO2; averaging 12–14 mL/g/min]) [6, 7, 8]. Therefore, correctly identifying high‐risk patients in the HFpEF population is a critical step in our current knowledge of the prognosis and clinical strategy development of HFpEF.

Recent findings have highlighted that pulmonary hypertension (PAH) and right ventricular (RV) dysfunction are pivotal players in the complex evolving framework of HFpEF syndrome and both are powerful predictors of outcomes [3, 9, 10, 11]. RV dysfunction is best assessed by cardiac magnetic resonance [12, 13]. However, in clinical practice, a single measure of echocardiography‐derived tricuspid annular plane systolic excursion (TAPSE) reflecting longitudinal right ventricular fiber shortening is often used to evaluate RV systolic function, which is feasible, repeatable, and highly predictive of adverse outcomes in a wide spectrum of HF cohorts [14, 15]. RV function is affected by afterload, such as pulmonary pressure [16]. Given this, the evaluation of RV function must be associated with an assessment of pulmonary pressures. Researchers defined this matching relationship between RV systolic force and afterload as RV‐pulmonary artery coupling (RVPAC). Pulmonary arterial systolic pressure (PASP) estimated noninvasively by Doppler echocardiography reflects the pulmonary pressure generated by RV contraction and is highly correlated with the pulmonary pressure detected by right heart catheterization [17, 18]. In recent years, the ratio of TAPSE to PASP derived by echocardiography, an indicator of RV longitudinal axis shortening and developed pressure in patients with HF, has been introduced as a noninvasive measurement of RV function and RVPAC, which has attracted wide attention [19, 20, 21]. However, it has not been adequately evaluated in HFpEF in detail.

Accordingly, we hypothesized that RVPAC may be used and proposed as an expression of key risk factors in patients with HFpEF. And the ratio between TAPSE (length) and PASP (developed pressure) may better disclose the prognosis compared with either variable in isolation.

## 2 Methods

### 2.1 Study Population

We retrospectively analyzed previously constructed data of 648 consecutive HFpEF patients, admitted to the cardiology department of a teaching hospital in Chongqing (China) between January 1, 2016 and January 1, 2017. All patients underwent a two‐dimensional echocardiographic/Doppler evaluation. Patients with HFpEF were selected according to the Chinese Guidelines for the diagnosis and treatment of Heart Failure in 2018 [22]. The diagnostic criteria for HFpEF are as follows: (1) presence of HF as defined by Framingham criteria, (2) left ventricular ejection fraction (LVEF) ≥ 50%, (3) NT‐proBNP > 125 pg/mL, and (3) transthoracic echocardiography meeting at least one of the following diagnostic criteria: (a) left ventricular hypertrophy and/or left atrial enlargement, and (b) ratio of early diastolic mitral inflow velocity (E) to early diastolic mitral annulus velocity (e′) ≥ 13 and/or mean value of e′ (interventricular septum and left ventricular lateral wall) < 9 cm/s. Exclusion criteria were: history of reduced LVEF < 40%, constrictive pericarditis, lack of data of the inferior vena cava and/or Doppler signal at the tricuspid regurgitation (TR) jet velocity to calculate the TAPSE/PASP ratio. Thus, the final cohort included 414 patients.

### 2.2 Data Sources

The following data were retrieved from the medical record: demographics, comorbidities, therapy, New York Heart Association (NYHA) functional class, NH2‐terminal pro‐brain‐type natriuretic peptide (NT‐pro‐BNP), length of stay, and outcome data. The admission value of all clinical measurements and laboratory values was defined as the first value recorded after admission. The time to death or readmission was recorded for all patients via hospital and outpatient medical chart review.

### 2.3 Echocardiographic Data

Chongqing Hospital of Traditional Chinese Medicine echocardiography data was queried, and the transthoracic echocardiography performed closest to the date of admission (either before or after) was identified. An echocardiographic assessment was performed using Philips IE33 (Philips Medical Systems, Netherlands) with a 1.0–5.0 MHz probe. One LVEF value for each patient was determined using a hierarchical approach: LVEF assessed by the biplane Simpson's rule was preferred, followed by two‐dimensional LVEF, or visually assessed when image quality was poor. TAPSE was measured using M‐mode according to the American Society of Echocardiography with the cursor optimally aligned along the direction of the tricuspid lateral annulus in the apical four‐chamber view [23]. TAPSE was measured as the peak excursion of the tricuspid annulus (millimeters) from the end of the diastole to the end‐systole. PASP was estimated by measuring the maximum continuous Doppler‐derived velocity of the TR jet. RV systolic pressure was determined from the TR jet velocity using the simplified Bernoulli equation, and combining this value with an estimate of the right atrial pressure by the diameter and collapsibility of the inferior vena cava that was added to the calculated gradient to yield PASP [23, 24]. Because subjects had no significant RV outflow tract or pulmonic valve obstruction, RV systolic pressure was considered equal to PASP [25]. RVPAC was approximated by calculating the ratio of TAPSE to echocardiographic‐derived PASP (i.e., TAPSE/PASP).

### 2.4 Blood Sampling Procedures and Assays

All patients had a measurement of plasma NT‐pro‐BNP (pg/mL). Venous blood samples were obtained after at least 30 min of rest and collected in tubes containing an EDTA buffer. They were immediately placed on ice and centrifuged at 4°C. Plasma samples were stored at 20°C until assay. The time between NT‐pro‐BNP and echo measurements was 2 ± 1 days. The instrument used is the multifunctional immunometer (model SSJ‐2) produced by Shenzhen Ruilai Company.

### 2.5 Follow‐Up and Outcomes

The follow‐up period for all patients was 5 years, with the first HF‐related recurrent hospitalization recorded. Follow‐up was terminated upon the patients' all‐cause death, confirmed through hospital and outpatient medical chart review. All‐cause death was selected as the primary outcome and the composite endpoint of all‐cause death or HF‐related recurrent hospitalization was selected as secondary outcome. HF‐related readmission was every hospitalization in which AHF or worsening HF was the main diagnosis at discharge. Only unplanned readmissions were included.

### 2.6 Statistical Analysis

Continuous and categorical data are reported as means ± SD and percentages. Comparison between the groups (survivors vs. nonsurvivors) was performed by unpaired *t*‐test or Mann–Whitney *U* test for continuous and *χ* ^2^ for categorical variables. Receiver operating characteristic (ROC) curve analysis was used to determine the optimal TAPSE, PASP, and TAPSE/PASP cutoff values for all‐cause death. The optimal cutoff for predicting all‐cause death was defined as the highest value of the Youden J index (sensitivity + specificity − 1) and the area under the curve was calculated. Linear regression analysis was used to assess the relationship between PASP (independent variable) and TAPSE (dependent variable) according to survivors versus nonsurvivors, free‐event versus endpoint event (HF‐related recurrent hospitalization or all‐cause death), and a TAPSE threshold of </≥ 16.5 mm. The optimal cutoff value for TAPSE/PASP was used for survival analysis in the present study. Kaplan–Meier analysis was used to assess survival or endpoint event rates across the optimal cutoff value for the TAPSE/PASP ratio. The log‐rank test determined statistical significance among the risk categories for all Kaplan–Meier analyses. A univariate Cox regression analysis for key clinical, laboratory, and echocardiographic variables was performed to determine endpoint event hazard ratios (HRs), reported with 95% confidence intervals (CIs). Variables found to be statistically significant in the univariate analysis were included as covariates in the multivariate Cox regression analysis. A two‐sided *p* < 0.05 was considered to be statistically significant for all analyses. Data were analyzed using the IBM SPSS Statistics software, V. 28.

## 3 Results

### 3.1 Patient Population

The study population included 414 patients with a mean age of 74.9 ± 11.0 years, out of which 170 (41.1%) were men. Table 1 provides the demographic, comorbidities, therapy, key clinical, biochemical, and echocardiographic characteristics of the studied cohort according to the primary endpoint status. The mean LVEF was 61.8 ± 7.1%, mean TAPSE 18.5 ± 3.6 mm, and mean PASP 34.4 ± 14.4 mm/Hg. Nonsurvivors (*n* = 20) were older with a more frequent history of end‐stage renal disease (ESRD), PAH, and less angiotensin‐converting enzyme inhibitors/angiotensin II receptor blocker (ACEI/ARB) prescriptions and had significantly higher NT‐pro‐BNP and NYHA functional class compared with survivors during the tracking period (*p* < 0.05) (Table 1). They also had significantly lower TAPSE, TAPSE/PASP and higher PASP, degree of TR compared with survivors (*p* < 0.0001) (Table 1). LVEF was numerically but not significantly lower in nonsurvivors. No substantial difference was observed for gender and hospital length of stay between survivors and nonsurvivors.

**Table 1:** Baseline characteristics of the patients according to survival status.

	Total population (*N* = 414)	Survivors (*N* = 394)	Nonsurvivors (*N* = 20)	*p* value
Age (years)	74.9 ± 11.0	74.6 ± 9.4	81.0 ± 8.2	0.0113
Male (%)	41.1	41.1	40.0	0.9211
Body mass index (kg/m^2^)	23.2 ± 2.4	23.1 ± 2.4	24.0 ± 2.9	0.095
Hospital length of stay (days)	10.0 ± 8.0	10.0 ± 4.6	13.0 ± 7.5	0.0910
Comorbidities, *n* (%)				
History of DM	127 (3.7)	122 (31.0)	5 (25.0)	0.5726
History of HTN	278 (67.1)	265 (67.3)	13 (65.0)	0.8338
History of ESRD	21 (5.1)	16 (4.1)	5 (25.0)	< 0.0001
History of CAD	225 (54.3)	213 (54.1)	12 (60.0)	0.6029
History of AF	158 (38.2)	148 (37.6)	10 (50.0)	0.2640
History of PAH	71 (17.1)	62 (15.7)	9 (45.0)	0.0007
History of VHD				
MS	13 (3.3)	12 (3.0)	1 (5.0)	0.4798
MR	16 (3.9)	14 (3.5)	2 (10)	0.1774
AS	11 (2.7)	10 (2.5)	1 (5.0)	0.4239
AR	9 (2.2)	9 (3.3)	0	1.0000
TR				< 0.0001
Mild of TR	256 (61.8)	254 (64.5)	2 (10.0)	
Moderate of TR	71 (17.2)	64 (16.2)	7 (35.0)	
Severe of TR	87 (21.0)	76 (19.3)	11 (55.0)	
NYHA functional class, *n* (%)				0.0286
I	18 (4.3)	18 (4.0)	0	
II	146 (35.3)	145 (37.0)	1 (5.0)	
III	171 (41.3)	165 (42.0)	6 (30.0)	
IV	79 (19.1)	66 (17.0)	13 (65.0)	
Therapy, *n* (%)				
ACEI/ARB	242 (58.5)	236 (59.9)	6 (30.0)	0.0081
ANRI	26 (6.3)	23 (5.8)	3 (15.0)	0.1227
Beta‐adrenergic receptor blockers	199 (48.1)	190 (48.2)	9 (45.0)	0.7784
Diuretic agents	346 (83.6)	328 (83.2)	18 (90.0)	0.5507
LVEF (%)	61.8 ± 7.1	61.9 ± 7.1	58.9 ± 6.8	0.0609
TAPSE (mm)	18.5 ± 3.6	18.7 ± 3.5	14.7 ± 3.6	< 0.0001
PASP (mmHg)	34.4 ± 14.4	33.7 ± 14.0	48.6 ± 16.2	< 0.0001
TAPSE/PASP (mm/mmHg)	0.64 ± 0.29	0.65 ± 0.29	0.33 ± 0.13	< 0.0001
NT‐pro‐BNP (pg/mL)	3474.6 ± 3493.7	3330.7 ± 3341.5	6310.4 ± 5048.0	0.0168

*Note:* Values are means ± SD.

Abbreviations: ACEI, angiotensin‐converting enzyme inhibitors; AF, atrial fibrillation; AR, aortic regurgitation; ARB, angiotensin II receptor blocker; ARNI, angiotensin receptor neprilysin inhibitor; AS, aortic stenosis; CAD, coronary artery disease; DM, diabetes mellitus; ESRD, end‐stage renal disease; HTN, hypertension; LVEF, left ventricular ejection fraction; MR, mitral regurgitation; MS, mitral stenosis; NT‐pro‐BNP, NH2‐terminal pro‐brain‐type natriuretic peptide; NYHA, New York Heart Association; PAH, pulmonary hypertension; PASP, pulmonary arterial systolic pressure; TAPSE, tricuspid annular plane systolic excursion; TR, tricuspid regurgitation; VHD, Valvular heart disease.

### 3.2 ROC Curve Analysis

**Figure 1:**
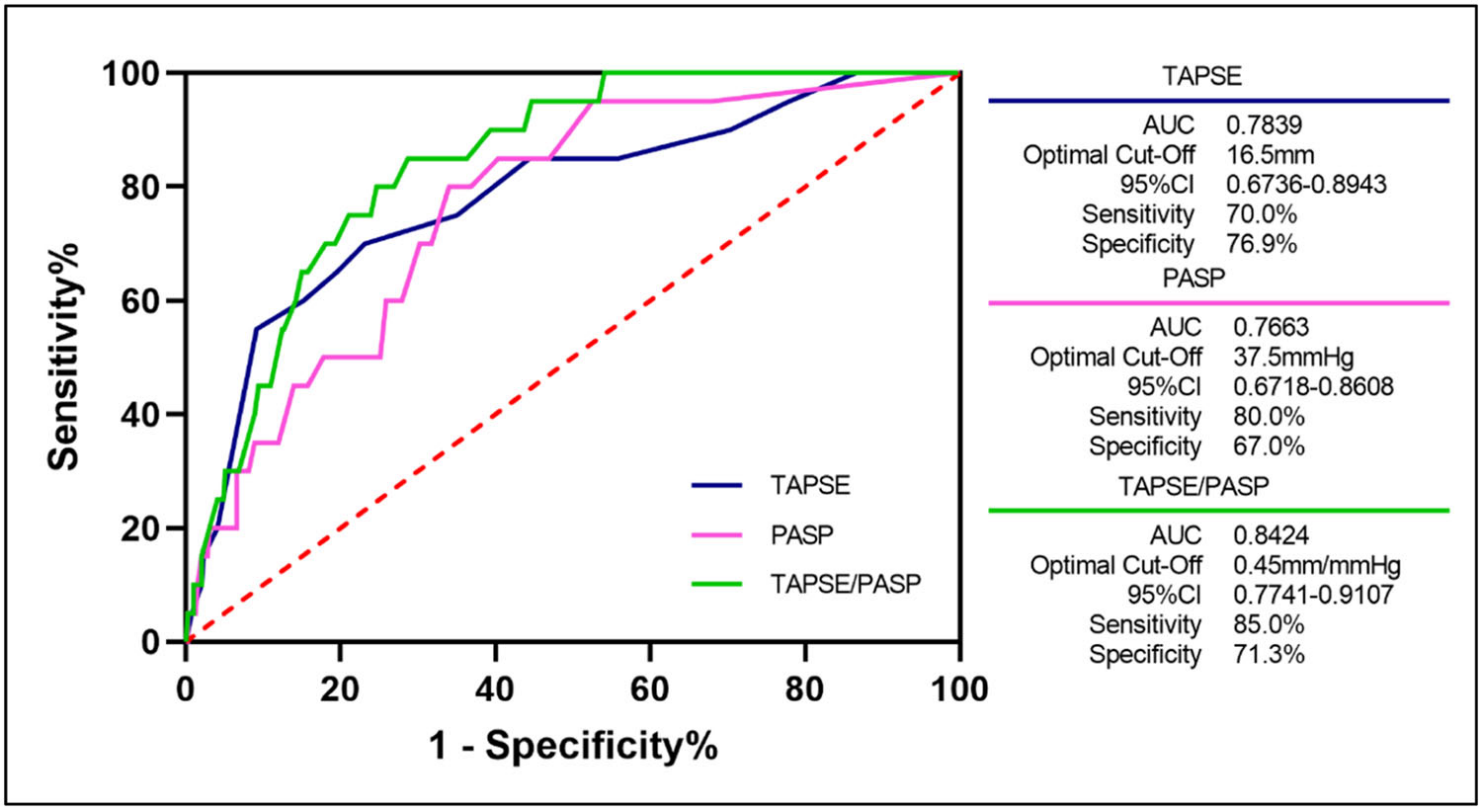
Receiver operating characteristic curve analysis for the relationship between right ventricular–pulmonary arterial coupling and its components with all‐cause death. The optimal cutoff of TAPSE was 16.5 mm, 37.5 mmHg for PASP, and 0.45 mm/mmHg for RVPAC, defined by the ratio of TAPSE to PASP. The ratio of TAPSE to PASP moderately predicted the study's primary outcome (area under the curve [AUC]: 0.8424).

ROC curves for the relationship between RVPAC and its components with the rate of all‐cause death (Figure 1). The optimal cutoff of TAPSE was 16.5 mm, 37.5 mmHg for PASP, and 0.45 mm/mmHg for RVPAC, defined by the ratio of TAPSE to PASP. The ratio of TAPSE to PASP moderately predicted primary outcome (area under the curve: 0.8424, 95% CI: 0.7741–0.9107, sensitivity 85.0%, specificity 71.3%, *p* < 0.0001).

### 3.3 TAPSE Versus PASP: Relationship and Ratio

**Figure 2:**
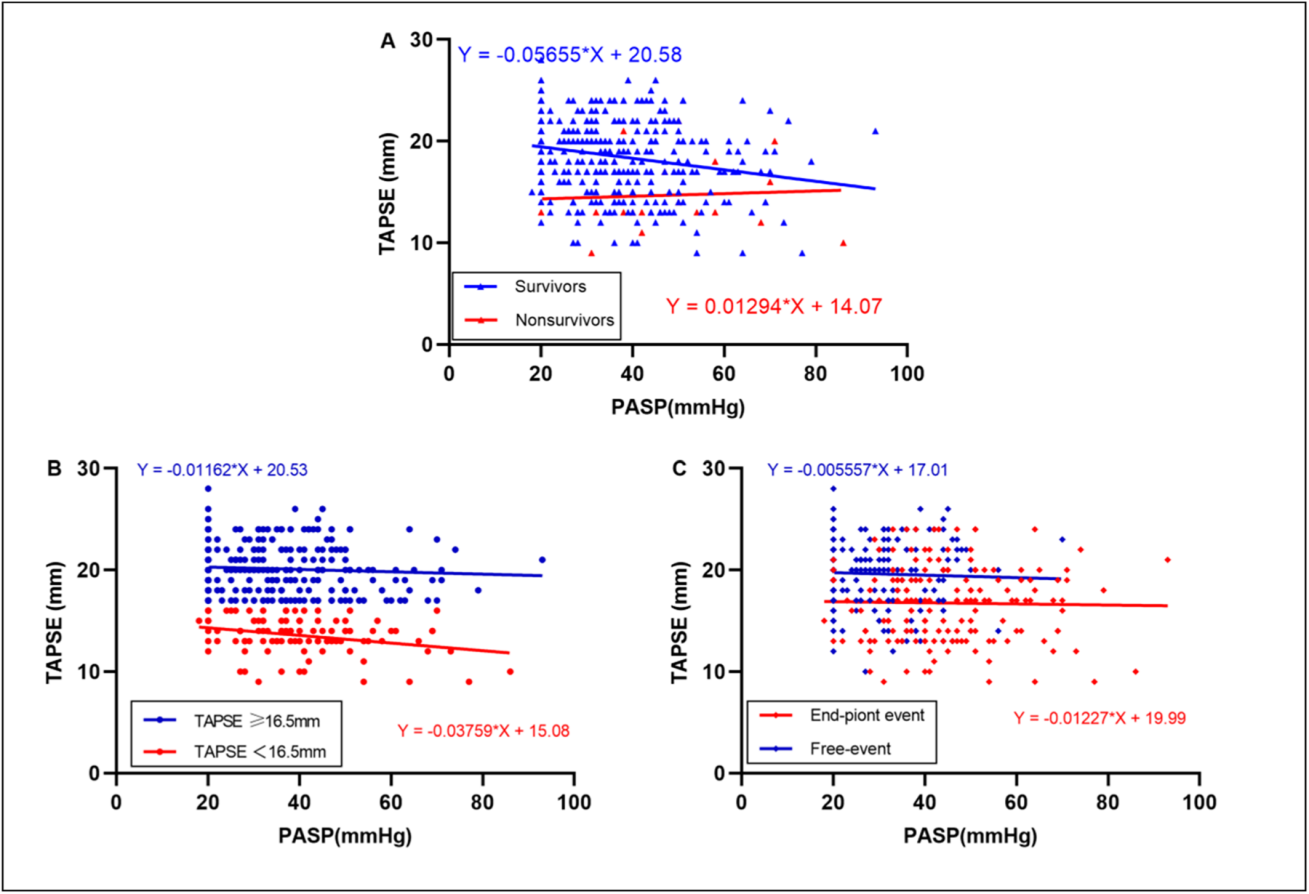
Plot of TPSE/PASP relationship according to survivors versus nonsurvivors (A), the optimal cutoff of TAPSE was 16.5 mm (B), and endpoint event (HF‐related recurrent hospitalization or all‐cause death) versus free‐event (C).

Figure 2 reports the TAPSE versus PASP relationship between survivors and nonsurvivors (A). When patients were separated according to TAPSE </≥ 16.5 mm, a downward shift occurred for the TAPSE < 16.5 mm group, with the slope of the regression significantly different (*p* = 0.0019) (B). Figure 2 also shows the regression line for free‐event and endpoint event (C), although the slope of the regression line was similar. The endpoint event exhibited a more unfavorable relationship, having higher PASP and lower TAPSE values, respectively. As shown in Figure S1, an inverse relationship was observed between TAPSE/PASP and NYHA functional class, with TAPSE/PASP decreasing as the severity of the NYHA functional class increased.

**Figure 3:**
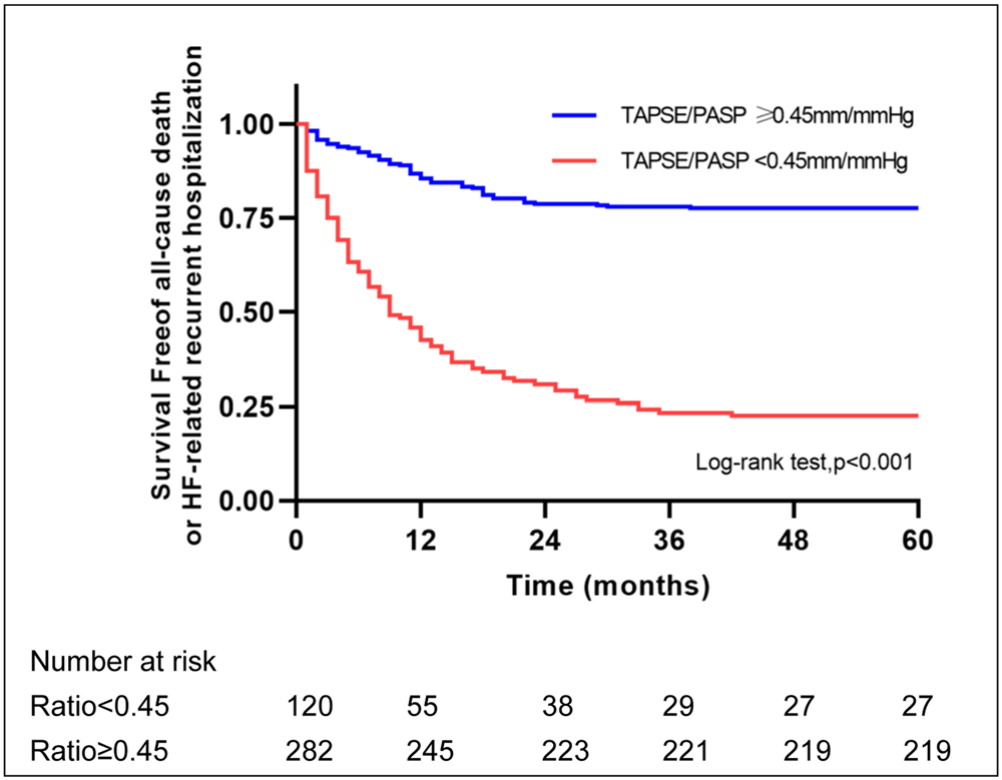
Kaplan–Meier curves for the composite endpoint of all‐cause death or HF‐related recurrent hospitalization, stratified by the optimal cutoff of TAPSE/PASP ratio.

The Kaplan–Meier survival curves according to the optimal cutoff value for TAPSE/PASP are reported in Figure 3, which illustrates that patients in the lower TAPSE/PASP ratio < 0.45 showed a higher risk of all‐cause death or HF‐related readmission (log‐rank *p* < 0.0001).

### 3.4 Univariate and Multivariate Analysis

Table 2 lists the univariate and multivariate Cox regression model for the prediction of all‐cause death. By univariate analysis, age, hospital length of stay, NT‐pro‐BNP, NYHA functional class, and all tissue Doppler echocardiography measures except LVEF were significant univariate predictors of all‐cause death in HFpEF. However, none of the univariate predictors were associated with all‐cause death after adjustment for gender and LVEF in multivariate analyses (*p* > 0.05). The results of the Cox regression model for the prediction of the endpoint event (HF‐related recurrent hospitalization or all‐cause death) are depicted in Table S1. In univariable analyses, age, hospital length of stay, NYHA functional class, TAPSE, PASP, TAPSE/PASP ratio, and NT‐pro‐BNP were all associated with the endpoint event. In multivariate analyses, including TAPSE/PASP as a continuous variable, the model (adjusting for gender) showed a significant, independent association of the TAPSE/PASP ratio with the endpoint event (HR: 0.006; 95% CI 0.001–0.057, *p* < 0.001) (Table S1). Of note, age and NT‐pro‐BNP also remained significantly associated with the endpoint event in this model.

**Table 2:** Cox regression model for the prediction of all‐cause death in HFpEF.

	Unadjusted HR	*p* value	Adjusted HR	*p* value
Sex	0.904 (0.387–2.316)	0.947		
Age	1.076 (1.017–1.139)	**0.011**	1.033 (0.979–1.089)	0.237
Hospital length of stay	1.096 (1.026–1.172)	**0.007**	1.050 (0.972–1.134)	0.218
NT‐pro‐BNP	1.00017 (1.00008–1.00026)	**< 0.001**	1.00006 (0.99994–1.00017)	0.337
LVEF	0.065 (0.883–1.004)	0.065		
NYHA functional class	5.084 (2.417–10.693)	**< 0.001**	1.678 (0.558–5.045)	0.357
TAPSE	0.753 (0.667–0.850)	**< 0.001**	0.965 (0.777–1.199)	0.747
PASP	1.053 (1.029–1.078)	**< 0.001**	0.984 (0.923–1.049)	0.622
TAPSE/PASP	0.001 (0.000–0.025)	**< 0.001**	0.070 (0.000–0.898)	0.228

*Note:* The multivariable analysis was adjusted for sex and LVEF. Bold values indicate *p* < 0.05.

Abbreviations: LVEF, left ventricular ejection fraction; NT‐pro‐BNP, NH2‐terminal pro‐brain‐type natriuretic peptide; NYHA, New York Heart Association; PASP, pulmonary arterial systolic pressure; TAPSE, tricuspid annular plane systolic excursion.

## 4 Discussion

We have presented a retrospective analysis of the TAPSE/PASP ratio in a large cohort of patients with HFpEF. Main study results include the following: (1) the TAPSE/PASP ratio is a determinant prognostic parameter in patients with HFpEF, which is independently associated with the composite endpoint of all‐cause death or HF‐related recurrent hospitalization; however, it is not an independent factor associated with all‐cause death; (2) TAPSE/PASP values decrease as NYHA functional class increases in patients with HFpEF; and (3) analyses with RVPAC provided greater prognostic value than the analysis of both entities separately.

HF is a complex clinical syndrome. Previous studies have shown that HFpEF accounts for approximately half of HF patients and is associated with poor quality of life, extensive utilization of medical resources, and premature death [4, 26]. In recent years, with the aging of the population, HFpEF has become the main form of HF, which has attracted the attention of scholars around the world [6]. In the clinical evaluation of patients with HFpEF, the assessment of prognosis and risk factors for death is still an important issue that needs to be solved urgently.

A large number of studies have confirmed that RV function has been a decisive factor in the prognosis of the HFpEF population [9, 27]. Previous studies mostly used a single measure, such as TAPSE, which considers only a single‐dimensional component of contractile function, to reflect RV systolic function [14]. However, a comprehensive assessment of the systolic function of the right heart should take into account the high sensitivity of the right heart to afterload. Fortunately, RVPAC, defined by the ratio of TAPSE to PASP in the present study, appropriately reflects the adaptation of the RV to changes in afterload, which is highly correlated with invasive PA compliance [28]. The TAPSE/PASP ratio obtained by echocardiographic measurement is more readily available than by other invasive and noninvasive RVPAC methods, such as the ratio of RV end‐systolic elastance/Pulmonary arterial elastance [29], the ratio of RV end‐systolic volume/stroke volume [30], the ratio of RV free wall strain/PASP [31], and so on. Further, we can even measure the TAPSE/PASP ratio at the bedside to evaluate RV function, even when the quality of the 2D echocardiographic image is poor, which provides a simple and quick way to assess prognosis. In addition, the TASPE/PASP ratio, as a substitute for RVPAC, has been widely used in various prognostic studies of diseases and has attracted more and more attention in recent years [32, 33].

As shown in this study, the comparative analysis between nonsurvivors and survivors showed that nonsurvivors always had higher PASP and lower TAPSE values than survivors. The regression line of the nonsurvivor distribution is below the regression of the survivors, indicating not only a lower TAPSE in the nonsurvivors at a given systolic load but also a decreased right ventricular systolic capacity in the nonsurvivors because the TAPSE versus PASP relationship had a steeper slope in TAPSE < 16.5 mm. Previous studies have confirmed that the TASPE/PASP ratio has a clear ability to classify disease severity and prognosis in HF patients [34, 35]. In a series of studies on the TAPSE/PASP ratio in patients with acute HFpEF, Gorter et al. found that the TAPSE/PASP ratio was not an independent predictor of atrial fibrillation (AF) in patients with HFpEF, even though AF was very common in the HFpEF population, confirming that TAPSE/PASP ratio measurement was not affected by AF [36]. In addition, Nakagawa et al., in a prospective study of 655 patients with acute HFpEF, confirmed that TAPSE/PASP is a strong prognostic indicator for decompensated HFpEF patients, especially the TAPSE/PASP ratio < 0.48, which significantly and independently predicted all‐cause death or the composite endpoint of all‐cause death and rehospitalization [34]. Our study further confirms these conclusions, as shown in the multivariate Cox regression model; RVPA uncoupling (i.e., the optimal truncation value of the TAPSE/PASP ratio < 0.45) was significantly independently associated with the composite endpoint of all‐cause death or HF‐related recurrent hospitalization. In addition, this study compared the coupling value with NYHA function class, and as expected, RVPAC in HFpEF patients decreased with an increase in NYHA function class, which is consistent with the results of Guazzi et al. [19], providing further evidence‐based evidence that RVPA uncoupling is independently associated with adverse outcomes in patients with HFpEF. Of course, some researchers used RVPAC indexes different from those obtained by echocardiography mentioned in our study to evaluate the prognosis of the HF population. Although similar conclusions were obtained that lower coupling values were related to worse prognosis, unfortunately, they did not distinguish HF population [30, 31]. Our study selected a clinically simple RVPAC evaluation method, focusing on the patients with HFpEF, and confirmed that the reduced TAPSE/PASP ratio (< 0.45) was a marker of significant RV dysfunction and poor outcomes in patients with HFpEF, which has certain reference value for guiding doctors' early clinical decision‐making. It is worth noting that this study and the cohort study of Nakagawa et al. [34, 37] from Asia (the cutoff value of TAPSE/PASP ratio of both was 0.48) compared with the cohort study of Guazzi et al. [19, 38] from Europe (the cutoff value of TAPSE/PASP ratio of both was 0.36) showed a higher TAPSE/PASP ratio, which may be due to the different basic conditions of Asians and Westerners themselves. From this point of view, whether the same TAPSE/PASP ratio has the same prognostic value in Asians and Westerners deserves further investigation. Anyway, the robust association between RV‐PA uncoupling (i.e., a low TAPSE/PASP ratio) and adverse outcomes suggests that interventions that improve coupling may improve outcomes.

Where does RVPAC's ability to predict adverse outcomes in HFpEF populations come from? It may be related to the following mechanisms. First is the effect of anatomical structure. The anatomy of the heart determines that both ventricles share myocardial muscle fibers and septum and that LV provides about 20%–40% of the contractile force to RV [16]. Although HFpEF is mainly characterized by LV diastolic dysfunction, there was evidence that HFpEF has LV systolic dysfunction and sub‐endocardium fibrosis [27]. It is not difficult to speculate that LV systolic dysfunction will decrease RV systolic force, and the fibrosis process affecting LV sub‐endocardium may also occur in RV. Therefore, these changes lead to RV systolic dysfunction in HFpEF patients (i.e., impaired longitudinal right ventricular systolic function, manifested as reduced TAPSE). The second is the impact of atrial fibrillation. Kotecha et al. observed that when HFpEF combined with AF, it increased right‐sided chamber dilatation and pulmonary artery pressure, leading to a decline in the overall function of the right heart [39]. The third is the influence of comorbidity. Some noncardiac comorbidities, such as renal insufficiency and so on, have adverse effects on myocardium through systemic chronic inflammation or endothelial function damage [27, 40]. Although these adverse impacts mainly focus on LV, they may also lead to the remodeling of pulmonary vessels and RV, considering the involvement of the systemic circulatory system.

Based on the possible mechanism mentioned above, we speculated that the change in the TAPSE/PASP ratio may indicate the degree of pulmonary vascular remodeling and RV remodeling, namely the degree of PH and RVD. Therefore, there is good reason to believe that TAPSE/PASP can serve as an alternative marker for RVPAC and provide a strong predictor of adverse outcomes in HFpEF populations.

## 5 Limitation

Several limitations of the study warrant comments. First, this is a single‐center retrospective analysis; missing data may affect the results and introduce bias and the conclusions need to be validated in other areas. In addition, we did not have invasive hemodynamic data to compare with noninvasive data, which limits our ability to make firm inferences. Finally, the fact that we only have explored RVPAC at rest may not completely reveal the effects of RVPAC on different states in the study population, considering the association between RVPAC and exercise capacity. Hence, larger multicenter prospective studies are needed to explore the optimal cutoff value and potential clinical prognostic value of RVPAC in HEpEF patients in different states.

## 6 Conclusion

In conclusion, the TAPSE/PASP ratio, as a substitute for RVPAC, is the expression of a key risk factor in HFpEF patients, which is independently associated with the composite endpoint of all‐cause death or HF‐related recurrent hospitalization. The evaluation of RVPAC was used to identify high‐risk patients with HFpEF and provide evidence for clinical decision‐making.

## Author Contributions

Study concept and design: Jia Wang and Ming Wang. Acquisition of data, analysis and interpretation of data statistical analysis: Jia Wang, Xiang Li, Jiahui Jiang, Ze Luo, Xilun Tan, and Ming Wang. Drafting of the manuscript: Jia Wang and Ming Wang. Critical revision of the manuscript and final approval of the version to be published: Jia Wang, Xiang Li, Jiahui Jiang, Ze Luo, Xilun Tan, Ruhua Ren, Tsutomu Fujita, Yoshifumi Kashima, Tetsuaki Li Tanimura, Ming Wang, and Chenhao Zhang. All authors agreed to be accountable for all aspects of the work in ensuring that questions related to the accuracy or integrity of any part of the work are appropriately investigated and resolved.

## Ethics Statement

The study conforms with the principle outlined in the Declaration of Helsinki and was approved by the Institutional Review Board of Chongqing Hospital of Traditional Chinese Medicine (Approval No.2022‐KY‐KS‐WJ) as posing minimal risk to patients, and was performed under a waiver of informed consent.

## Conflicts of Interest

The authors declare no conflicts of interest.

## Supporting information

Supporting information.

Supporting information.

## Data Availability

Due to the nature of this research, participants of this study did not agree for their data to be shared publicly, so supporting data is not available.
