# Supplementary material for: Right Ventricular–Pulmonary Arterial Coupling and Outcome in Heart Failure With Preserved Ejection Fraction
Source: Clin Cardiol. 2024 Jul 16;47(7):e24308. doi: 10.1002/clc.24308 (PMC11249816; doi:10.1002/clc.24308)

**Supplementary Figure 1**

Average TAPSE/PASP distribution based on New York Heart Association (NYHA) functional class. ns, *P*≥0.05; *****P*<0.0001.


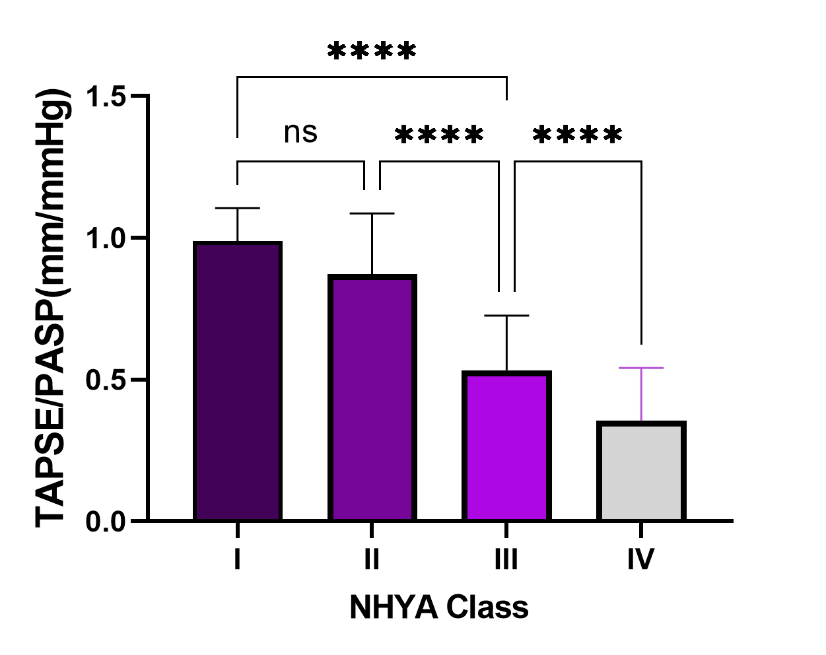

Supplement: Supplementary file 1 — Supporting information. [file CLC-47-e24308-s002.docx]
