# Supplementary material for: Right Ventricular–Pulmonary Arterial Coupling and Outcome in Heart Failure With Preserved Ejection Fraction
Source: Clin Cardiol. 2024 Jul 16;47(7):e24308. doi: 10.1002/clc.24308 (PMC11249816; doi:10.1002/clc.24308)
Supplement: Supplementary file 2 — Supporting information. [file CLC-47-e24308-s001.docx]

**Supplementary Table 1**

Cox regression model for the prediction of the composite endpoint of all-cause death or HF-related recurrent hospitalization in HFpEF

|  | **Unadjusted HR** | ***P* value** | **Adjusted HR** | ***P* value** |
| --- | --- | --- | --- | --- |
| Sex | 0.928(0.682-1.264) | 0.637 |  |  |
| Age | 1.042(1.025-1.060) | **<0.001** | 1.019(1.003-1.036) | **0.018** |
| Hospital length of stay | 1.063(1.035-1.091) | **<0.001** | 1.029(0.999-1.059) | 0.054 |
| NT-pro-BNP | 1.00013  (1.00010-1.00017) | **<0.001** | 1.00006  (1.00001-1.00011) | **0.032** |
| LVEF | 0.967(0.946-0.988) | **0.002** | 1.023(0.998-1.050） | 0.075 |
| NYHA functional class | 3.418(2.752-4.245) | **<0.001** | 1.343(0.960-1.880) | 0.085 |
| TAPSE | 0.835(0.801-0.871) | **<0.001** | 1.025(0.953-1.103) | 0.506 |
| PASP | 1.060(1.051-1.070) | **<0.001** | 0.992(0.969-1.016) | 0.533 |
| TAPSE/PASP | 0.005(0.002-0.012) | **<0.001** | 0.006(0.001-0.057) | **<0.001** |

The multivariable analysis was adjusted for sex.
